# On Typhoid Affection

**Published:** 1822-10

**Authors:** 


### ART. III.—*On Typhoid Affection.* By Dr. KINGLAKE.

IT is a circumstance fraught with vast practical evil, that the true nature of typhoid affection is not sufficiently regarded to determine in what it essentially consists. Febrile diseases of all descriptions sooner or later assume the typhoid character, according to either the kind or degree of visceral oppression that may arise. Fevers of every denomination are in their onset radically similar, inasmuch as they are, in all their diversities, the common effect of immediate excitement. Various parts of the system are apt to become the seat or source of the morbid excitement, that at once originates and continues febrile disease. If the natural exertion of vital power that constitutes health be disturbed, the effect will be variously shown. The change from the healthful condition deranges the natural harmonies of life, and induces strong efforts to regain the lost calmness. The conflict thus arising is febrile agitation, nor can it cease until either the healthy state or a condition approaching to it be restored. If the main seat of this disordered action be in a part essential to life, the diseased function of that particular part, as well as the sympathetic connexion subsisting between it and other portions of the frame, will be disturbed, and will co-operate in producing a greater or less degree of morbid excitement. The notion of diseased action being inflammatory or not, can only refer to the degree of the existing affection.

If the excitement go the length of inducing increased action [illegible] the diseased part, it may reach the extent of inflammato[illegible] violence or not, according to the natural and morbid circumstances of the case. Essentially considered, the ground-work of the disease is similar, and is only varied in external character by the modifying influence of collateral circumstances.

Febrile symptoms, under whatever name designated, denote increased action of vital power. What has induced this departure from the quietude of health? A correct answer to this question would be an accurate definition of the existing disease. All the circumstances of a morbid affection may not be perceived, and, if they were, they would be found to be rather the incidental filling-up, than the rudimental conditions of the diseased state. When distinctions are visionarily drawn between objects in which there is no real difference, erroneous conclusions are apt to be the result. It is of importance to guard against such gratuitous inferences, lest the judgment might be incorrectly persuaded by them. It should not be forgotten, but be kept incessantly in view, in speculating on the nature and cause of typhoid and other febrile forms of disease, that vital organs, of more or less moment to life, are fundamentally involved in these ailments. It is generally to be perceived, on a thoughtful review of the attending symptoms, where the source of the disease is, and what circumstances in the case may appear to be original, and what sympathetic. To distinguish accurately between the part in which the disease exists, and others to which its morbid influence may have been sympathetically extended, is of the utmost consequence in the curative treatment.

The various circumstances that may be unfriendly to health,—such as change of temperature, excessive fatigue, insufficient sustenance as well as immoderate indulgence, mental anxiety, breathing an atmosphere either contaminated with unwholesome effluvia, or impregnated with substances that mechanically displace the quantity of oxygenous air that is necessary for animal health,—these and other noxious agents may derange that equal distribution of vital action and of the circulating fluids on which the salutary state of life depends, and may occasion diseases varying in form and degree, according to the particular influence that may have induced them. The origin, as well as the progress, of the diseased action will determine the precise nature and character of the occurring affection. If it be decidedly inflammatory, it is not regarded as typhoid, but treated as disease of over-excitement, and is speedily subdued. When the symptoms are less obviously of the inflammatory type, in as far as they are less acute and not evidently referrible to any particular organ, and attended with extreme debility, then it is considered as typhoid, and may appear to require rather sustaining than depleting means of relief.

It would be a great practical advantage to assimilate the treatment of what are called inflammatory and typhous fevers, inasmuch as they may be justly held to be radically alike. In both instances, the great danger consists in the safety of the visceral part in which the disease may have arisen, as well as in the sympathetic extension of its violence, more or less generally, throughout the system. The prevailing debility in the more evidently inflammatory, as well as in typhoid affection, is induced by depression, not by exhaustion, of strength. The healthy tone of life, on which both the sense and power of strength depend, is overthrown, and kept as it were in a state of abeyance during the disconcerting influence of disease. The calm, equal beat of arterial action, that denotes the condition of health, in all cases of debility, whether real or apparent only, is hurried into the agitated rapidity that attends febrile disease. This is, in reality, exhausting to the natural strength, and would, by long continuance, destructively waste the powers of life. This morbid excitement is a re-active effort made by vital power against the oppressive injury of vascular fulness or congestion in the affected organ. If this organ be the brain, the deranging influence of its disordered function over the whole frame will be strongly marked by pains, either stationary or fugitive, stimulating or torpifying, according to the degree and effects of the prevailing affection. The brain, from being a more sentient organ than any other viscus, is more susceptible of being hurtfully oppressed than any other part of the frame; and, although every natural provision is made for its protection in the tortuous and slow transmission of the circulating fluids to it, yet it may perhaps be justly regarded as giving occasion to more febrile disease, and that too of the worst character, than any other part, or even than the whole of the rest of the system.

In visceral disease, the attendant danger must be estimated by the nature and importance of the function that is disturbed, both in respect to itself and to the sympathetic influence which it may have in disordering other parts. Between the brain and every part of the frame, the most sensitive and prompt sympathies subsist. These will quickly share in its disease, and will impart to it the universality, complexity, and inveteracy, usually characterising febrile affection of the brain, or what may be appropriately phrased cerebral fever. The lungs, heart, liver, and intestines, together with all the various textures of the human body, particularly the muscular, ligamentous, and tendinous, may severally originate febrile affection, which may be prolonged into typhoid disease. It is, therefore, very material to the duration of suffering, as well as to the eventual safety of life, to shorten the continuance of febrile ailment, whatever part may either have commenced it, or may have become particularly oppressed in its progress. Protracted disease additionally disorders the part in which it may be especially situated; it vitiates the innate excitability, unfits it for its healthy function, and tends so to disorganize its structure as at length to incapacitate it for resuming its natural office. However, therefore, diseases may be circumstanced, and whatever speculations may be held concerning them, no question can be raised as to the propriety, nay urgency, of curing them with all possible despatch.

The relief of disease is the final and only useful object of all practical researches and theoretical considerations on the subject. To know the means for effecting this great intention is the desideratum, and they should be assiduously and unceasingly applied for the early and effectual accomplishment of that salutary purpose. In what do the curative means consist? The answer should explain the most appropriate and efficient treatment, and it will remain for the experience and judgment of medical practitioners to determine the admissibility, and to appreciate the practical advantage, of the proposed mode of remedy.

As before suggested, it would appear to be incontrovertibly true that febrile diseases of all descriptions are radically similar, differing only in form and degree; in which view of the subject, typhous fever cannot be justly considered as essentially varying from the aspect of affection that unequivocally assumes the highly inflammatory character. In typhoid affections, or in those morbid appearances in which immoderate excitement is apparently kept under by visceral congestion and venous plethora, the contractile power of the sanguiferous system is diminished by excessive distention; nor can it be relieved and rendered duly active, but by removing the plethoric and disabling fulness. On this being effected, increased exertion is restored to the contractile power which had been oppressed, and salutary energy is imparted to the various vital functions of the animal economy. In the more active form of inflammatory excitement, the contractile power of the heart and arteries is in full exertion, which gives it the character of extreme morbid violence. This is what may be regarded as active inflammation, in contradistinction to that which is called the passive, or chronic, form of that state. The real difference between the nominal conditions of active and passive inflammation, is that the contractile power of the arterial system is, in the former, freely and immoderately exerted; whilst, in the latter, it is too much oppressed and disabled by the torpifying influence of excessive distention, arising from congestive and plethoric fulness, to admit of being salutarily acted on. Active inflammation, therefore, agreeably to this distinction, is an undisguised complaint, too manifestly clear in its nature to be misunderstood; whilst the passive form of inflammatory disease is obscured and buried beneath the delusive mists of comparative inaction and sinking debility. The appropriate treatment in the former case is too evident to be mistaken; in the latter, much practical anxiety and doubt attend its management.

It would be of the highest importance in treating febrile affections of the actively inflammatory, and more especially those of the passive or typhoid type, to keep in view the immoderate excitement that lies at the root of these seemingly opposite diseases, to regard the one as serious for its exhausting and disorganizing influence, and the other as more insidiously dangerous, by depressing, tramelling, and, finally, extinguishing, as it were, the active powers of life. It is only in instances in which the nutritive processes of life have been interrupted, and nearly abrogated, by deficient sustenance, whether from insufficient aliment taken into the stomach, from indigestion, or from its obstructed transmission into the system through the mesenteric glands, that vital power can be at so low an ebb as to be incapable of hurtful excitement. In such circumstances, mental exhaustion is at hand, and could only be obviated by sustaining the sinking powers of life. These cases of sheer exhaustion are rare, and perhaps never occur but in the closing stages of maladies of long duration, where vital power is not viscerally depressed and lowered in its general energies, but drained and worn out by protracted disease.

Febrile affections are not, either at their commencement or in their more advanced progress, so connected with that debile or exhausted state of vital power, as to endanger, from that cause, an immediate extinction of life. The capability for being excited in these cases is greatly increased, rather than diminished, by the stimulating agency of disease; and this power is tenaciously retained, and more or less energetically developed, in the re-active exertions attending the febrile conflict. In the highly excited or excitable state invariably accompanying the earlier stages of febrile disease, what is there that admits of being radically distinguished from inflammatory excitement? The conditions are similar, but exhibited under the various aspects of a free and of an oppressed action of vital power. Extreme debility is no decided proof of exhausted power: it indicates that it is so clogged and depressed as to be incapable of acting with healthful energy. Nor is a frail, tremulous state of arterial action an infallible sign of deficient vital power: it shows that the energies of life are cramped and confined, and cannot become duly active without the removal of the oppressive impediment to the natural exertion. Neither petechiæ, nor vibices, nor bloody effusions in the intestines, in typhoid affections, are unerring indications of either a dying prostration of strength, or of the existence of putridity. They are, on the contrary, often denotations of immoderate excitement, having urged the red or globular part of the blood beyond the natural bounds of the circulation, forcing it into the narrow areas of the minuter division of the arterial system, such as the exhalent and capillary vessels, and sometimes into the interstitial spaces afforded by the cellular cavities.

The prevailing notion of putrescency of the circulating fluids is much too inconsiderately adopted. It is an apprehension without proof, and a condition which, in fact, could not exist. It would be incompatible with life, and never occurs but in the decomposition that necessarily arises after death. The blood that is drawn in the petechial, dysenteric, and other aggravated circumstances of typhous disease, is generally found to be dense, largely crassamentous, and often to exhibit a surface of coagulable lymph. These facts evince that the ideal putrefaction is not real, and that immoderate excitement, and not debility, is the cause of the prevailing disease.

Typhoid affections, of whatever particular denomination or symptoms, should be treated under a guarded reference to the state of the visceral organs. The leading inquiry should be, what vital organ is especially or disproportionately affected? If it be the brain, the importance of its function, as the sentient and grand energizing organ of the whole frame, demands early and efficient relief. If it be any other viscus, or, which is very common, if there be an intermixture of disease between two or more visceral parts, the case becomes proportionately more complicated, and requires additional promptitude in the removal.

The first step in the curative treatment should be to draw blood. The quantity to be taken must be determined by the greater or less severity of the concomitant symptoms. Different opinions have been held respecting the propriety of large bleeding at the commencement, with a view to breaking the force of the fever in its onset, and thereby to arrest its progress and bring it to an early termination. This is a controverted point, and its correctness would appear to be justly questionable. The visceral oppressions and partial plethoras existing in typhoid affections so distend and burthen vascular contractility, as to render it too torpid for the immediate action that is necessary to adapt the sanguiferous vessels to the diminished contents resulting from a very large bleeding, which incapacity may be followed by increased depression and final extinction of vital power. After the infliction of stunning or paralizing injury on the brain by external violence, arterial action is often in the depressed tremulous state in which it occurs from visceral, particularly cerebral, oppression from febrile disease: under such circumstances, to draw a large quantity of blood would be to destroy. It would have the effect of abstracting from the heart and arteries the stimulus of distention, without which life could not endure for a moment. But a small bleeding, in such cases, will cause an increased exertion to be made of the vascular contractility, which would at once quicken the motion of the circulating fluids and relieve partial oppressions. It would appear, therefore, to be the safest practice to begin with the removal of about eight ounces of blood, and to repeat it every eight hours until relief be obtained. A periodical bleeding to the extent mentioned, in two or three instances, will generally be found sufficient. The blood will be commonly dense, and will assume a buffy aspect. Relief almost invariably follows the first bleeding, so as to prove that the measure of direct vascular depletion by blood-bleeding is vindicable and salutary. Immediately after bleeding, the bowels should be freely moved by purgative medicine. It is not of much consequence what articles are employed to evacuate the intestines, provided the effect be fully produced. Infusion of senna saturated with sulphate of magnesia, or any other of the neutral salts, and occasionally quickened with a suitable proportion of powder of jalap, will answer the purpose sufficiently well. If the biliary secretion should be deficient, or its product be not freely transmitted to the duodenum, calomel and jalap may be interposed with advantage. But the desideratum in typhoid disease is an active and transmitting exertion of the peristaltic power of the intestines. This soluble state of the bowels at once obviates an hurtful source of irritation from an offensive quantity and quality of secreted and undigested or residual feculencies, and further aids by relieving other parts of the system of an oppressive weight of circulating fluids, by occasioning an additional flow of them on the intestinal canal. Fully to induce this beneficial determination, and to sustain a due action of the peristaltic or transmitting power of the intestines, opening medicine should be given, pending the more oppressive symptoms of typhous disease, at least once in twenty-four hours, and be repeated, if necessary, at proper intervals during that period, until ample discharges be obtained. A distended and renitent abdomen indicates insufficient contraction of the intestines, and should be removed and obviated by purgative excitement.

Dilution is of great importance in febrile diseases of every description, as well to attenuate and reduce the saline and acrid quality of the circulating fluids, as to enable them to flow equally over the whole frame, to support the various secreting processes, and probably to detach and carry off redundant heat and fluidity by cutaneous exhalation. For this purpose, farinaceous and mucilaginous liquids should be preferred,—such as barley-water, thin oat-gruel, infusion of bread, aqueous solution of gum-arabic: water slightly acidulated with vegetable acids in cases of high excitement, and also with those of the mineral kind, in circumstances of great exhaustion, would be desirable. In a similar intention, saline medicine, compounded of a neutralized combination of acid and alkali, and properly diluted with water, may be advantageously given.

Dietetic support, during the high conflict of fever, should not be carried the length of increasing the prevailing morbid excitement. It should consist chiefly of vegetable juices, extracted either by decoction or infusion, in the form of broth or tea. The animal juices are in general too stimulating in the early stages of typhous fever. In the advanced period of the disease, they may recruit diminished strength, and obviate increasing debility. Milk and eggs may be likewise resorted to, for the purpose of affording support to declining strength, in small quantities, at a time and at intervals sufficiently long to prevent either oppression at the stomach or the occurrence of an undigesting acetous fermentation.

Blistering in the neighbourhood of an affected vital organ, and the inside of the lower extremities, just below the calves of the legs, may benefit, both in a derivant and excitant intention, in protracted and sinking states of typhous fever. Fomenting the lower extremities, by means of flannel wrung out of hot water, renewed about every five minutes, and continued for half an hour at a time, repeating it every four or six hours,—afterwards rubbing the bathed parts quite dry, and wrapping them up in warm flannel,—often affords much relief, by at once deriving from the visceral parts and promoting an equal distribution of the circulating fluids over the frame.

Friction over the region of the stomach, and on the lower extremities, with a flesh-brush, carried the length of inducing efflorescence on the skin, may be usefully employed. Leeching or cupping near a suffering vital organ may be advisable under circumstances of arterial debility, that would forbid general bleeding. Sponging the whole surface with cold water when the skin is dry and hot, and with tepid when moist and cool, is a gratifying and serviceable practice.

Sufficient ventilation to admit of an unobstructed renewal of fresh air, should be regarded as indispensably necessary in the management of typhous affection. A moderate degree of heat should be preferred, and be as uniformly sustained as possible. Bed and body linen should be often changed, and the alvine and urinary discharges should not be suffered to remain in the patient's apartment. All unnecessary bodily exertion, particularly conversation, should be avoided, and mental tranquillity should be strictly enjoined.

The successful treatment of typhous fever will greatly depend on the concurrent influence of the evacuant, dietetic, and domestic assistances, that are required. The morbid excitement should be speedily reduced, lest congestive, inflammatory, disorganizing, and even gangrenous, mischief be induced. Protracted ailment is fraught with risk, and incidental danger can only be obviated by prompt and effectual remedy. Diseases of all kinds have a tendency to get worse, if not soon relieved. The re-active efforts of vital power against the inroad of disease often succeed in effecting recovery. The resources of nature are generally equal to the morbid conflict, and, when required, they may be eked out and sustained by appropriate remedies. Natural power had better be left to its own exertion, than be perverted and mis-directed by inapplicable modes of assistance.

Correct theory of the nature of disease suggests the most apposite and efficacious treatment. No scheme of remedy can be instituted without some sort of pathological speculation, and the whole difference between beneficial and injurious practice consists in the truth or fallacy of the speculation that may be held. Recoveries from typhous fever happen under opposite modes of treatment, which would go to prove that these instances were of a recoverable character, and would have terminated favourably had they been left to a natural course. But when mortal events occur under one mode of management, and not under that which may be founded on an opposite view of the nature of the disease, it is just to infer that the medicinal agency in the one case has been either pernicious or inefficient, and in the other remedial. Theories of diseases that are salutarily applicable to practice, must rest on conclusive facts. It is on a scrupulous induction from this only legitimate source of information that practical medicine can be securely founded. On this basis it must succeed to a given extent, and, though it may not be infallible, it will furnish all the aid which existing circumstances will permit.

*Taunton; July 25th,* 1822.

